# Visual analysis of allergic rhinitis in children based on web of science and CiteSpace software

**DOI:** 10.3389/fped.2022.911293

**Published:** 2022-09-28

**Authors:** Fang Liu, Na Chen, Rui Wang, Lei Zhang, Youwei Li

**Affiliations:** ^1^Department of Otolaryngology, Beijing Rehabilitation Hospital, Capital Medical University, Beijing, China; ^2^Department of Radiology, Beijing Rehabilitation Hospital, Capital Medical University, Beijing, China

**Keywords:** visualization analysis, allergic rhinitis, children, web of science, CiteSpace

## Abstract

**Background:**

In recent years, Allergic Rhinitis (AR) in children has caused widespread public concern. However, there are few studies concerning the overall trends in AR research in children based on bibliometric analysis.

**Objective:**

This study aims to explore hotspots and emerging trends in AR in children.

**Methods:**

The relevant publications were searched for in the Web of Science (WOS) Core Collection on December 31, 2021. The searched studies were exported to CiteSpace and Microsoft Excel for further visualized analysis.

**Results:**

In total, 649 articles were included. The number of publications related to AR in children has increased steadily in the last 20 years. Giorgio Ciprandi from Italy has the most articles and the leading countries were China and the USA. Guangzhou Medical University in China and Hallym University in Korea were the institutions with the most articles. The high-frequency keywords included AR, asthma, children, prevalence, and symptoms. Pathogenesis, comorbidity, epidemiology, symptoms, and therapy of AR in children are research hotspots.

**Conclusion:**

Over the past 20 years, research on AR in children has gradually improved. Visualization analysis indicates that pathogenesis, comorbidity, epidemiology, symptoms, and therapy are research hotspots, and immunotherapy and severity are probably the main research directions.

## Introduction

Allergic rhinitis (AR) is a non-infectious chronic inflammatory disease of nasal mucosa mediated by IgE after exposure to allergens (1) and characterized by clinical symptoms including nasal congestion, rhinorrhea, paroxysmal sneezing, or itching of the nose (2). The incidence of AR has been increasing for the last 20 years around the world and has a prevalence of up to 40% (3) in children. A survey of adults with nasal allergies in the United States estimated that about 14% of adults are diagnosed with AR. Almost 80% of individuals develop symptoms of AR before 20 years old, with 40% of patients becoming symptomatic by 6 years old (4, 5). AR has the potential to lead to asthma and OSAHS (Obstructive Sleep Apnea-Hypopnea Syndrome). The obvious link between AR and asthma has been confirmed in a World Health Organization (WHO) report in 2016, with approximately 30% of patients with rhinitis developing asthma and up to 80% of patients with asthma having nasal symptoms (6). AR affects the sleep, study, and quality of life of patients under 18 years old and even increases the economic burdens on the family.

Currently, the management of AR is environmental control, immunotherapy, and pharmacotherapy. At present, a very important part of the clinical treatment of AR in children is to avoid exposure to allergens, but this is often difficult to achieve in real life. Therefore, drug therapy remains the mainstay of treatment, which includes antihistamines, mast cell membrane stabilizers, corticosteroids, decongestants, anticholinergics, and leukotriene antagonists. Among these, antihistamines and corticosteroids are the main pharmacologic therapies for AR, and several of them have been approved for use in children (7). Modern medicine is effective in the treatment of AR in children, but the effect is difficult to maintain long-term. Studies have shown that pharmacologic treatment has a high recurrence rate and side effects (8). AR is an allergic disease of nasal mucosa mediated by immunoglobulin E (Ig E); therefore, immunotherapy is also one of the main means of clinical treatment of AR in children. There is no simple treatment that can fundamentally cure AR in children at present. Therefore, creating a summary of the *status quo* and analyzing the hotspots and emerging trends in AR research in children is urgent.

CiteSpace (5.8.R4), (9) developed by Dr. Chen Chaomei from Drexel University, is an application based on Java for data analysis. It is a good tool for bibliometric and visualization analysis of literature and has been widely used to summarize the hotspots and research trends of various fields in medicine (10–14). However, no visualization study on AR in children has been reported.

In this study, CiteSpace was used to analyze the publications related to AR in children from 2002 to 2021. The study aimed to explore the hotspots and emerging trends in the field.

## Materials and methods

### Search strategy

The relevant publications were searched for in the Web of Science (WOS) Core Collection including SCI-EXPANDED, SSCI, A&HCI, CPCI-S, CPCI-SSH, BKCI-S, BKCI-SSH, ESCI, CCR-EXPANDED, and IC on 31 December 2021. The following search strategy was used: TS = (adolescent or youth or teen or child or minors or children or childhood or infant) AND TS = (“allergic rhinitis” OR “hay fever”). Restrictions: (1) Languages (English); (2) Document types (article); and (3) Timespan (2002∼2021).

Studies were considered eligible only if the study population was limited to children diagnosed with AR based on the family history, typical allergic history, clinical presentation, and skin-prick tests (SPTs), or serum allergen-specific IgE. The exclusion criteria for studies were as follows: (1) statistical data were incomplete; (2) review papers and case reports; (3) did not have full-text available or repeated publications.

The articles that met the inclusion criteria, and did not meet the exclusion criteria, were used in this analysis. The articles were screened by two reviewers independently. If a disagreement between two reviewers appeared, a consensus was reached by the third reviewer.

### Bibliometric analysis

The selected studies were downloaded and exported to CiteSpace (5.8.R4) and Microsoft Excel (2016) for further analysis. Each study included a full record and all cited references. A visualization analysis including annual publications, authors, institutions, countries, keywords, and keywords with the strongest citation bursts was carried out by CiteSpace. The parameters of CiteSpace were as follows: timespan (2002∼2021), time slice (1 year), selection criteria (Top 50), and visualization (cluster view-static and show merged network). Microsoft Excel was used to draw the line graph showing the trend of published articles by year.

Ethical approval was not applicable for the present study.

## Results

### Numbers of publications by years

**FIGURE 1:**
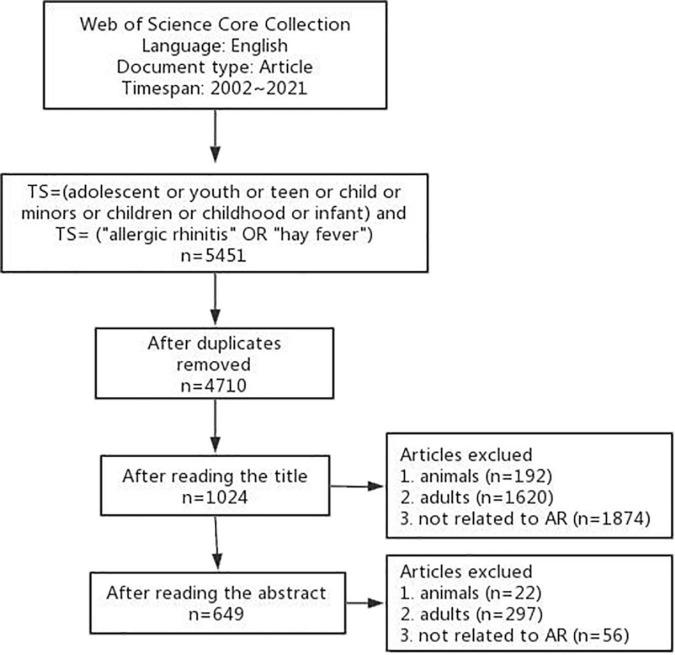
Flowchart of the search process for included articles.

In the past 20 years, a total of 5,451 articles in English were retrieved by the initial search. After removing the duplicates, 4,710 articles were left. The title of each article was reviewed by two reviewers, and 3,686 articles were excluded. For uncertain articles, the abstract was read by the reviewers. After this step, 375 articles were removed. Finally, 649 articles were included for further analysis. The flowchart of the search process is shown in Figure 1.

**FIGURE 2:**
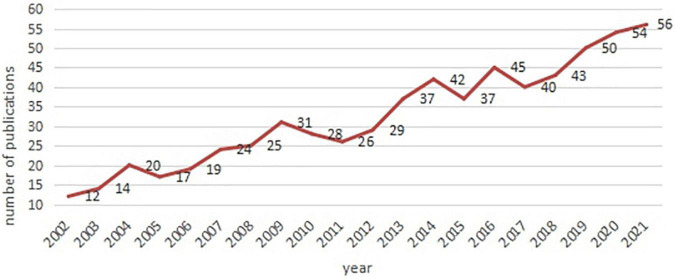
Trend of numbers of publications on AR in children by years.

The annual number of articles published in each field shows the development trend. The line graph (Figure 2) shows that the number of publications related to AR in children has increased over the past 20 years with a steady growth rate. The first English article on “AR in Children” in the WOS was published by Edwards (15) in.

### Analysis of authors

**FIGURE 3:**
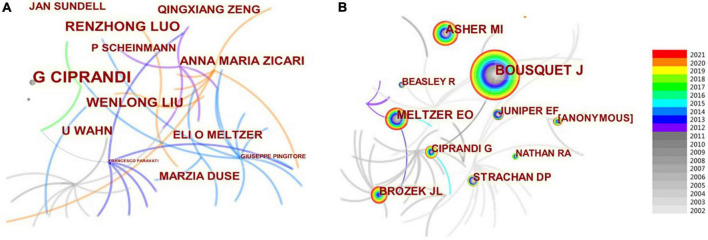
Relevant map of authors on AR research in children. **(A)** Author map. **(B)** Co-cited author map.

In the past 20 years, a total of about 3,299 authors have published studies on AR in children. In the author’s collaboration network map (Figure 3A), one node represents one author, and the size of a node stands for the number of articles published by the author. Gray nodes represent earlier published studies (2002∼2011), while colorful nodes represent studies published in the last 10 years. Many authors published only one or two articles each. The top 10 most active authors are shown in Table 1. Giorgio Ciprandi from Italy ranked first with a total of 19 publications, followed by Renzhong Luo (*n* = 11) and Wenlong Liu (*n* = 10) from China, U. Wahn (*n* = 8) from Germany, and Anna Maria Zicari (*n* = 8) from Italy.

**TABLE 1:** Top 10 active authors from 2002 to 2021.

No.	Author	Counts	No.	Author	Counts
1	Giorgio Ciprandi	19	6	Marzia Duse	7
2	Renzhong Luo	11	7	Qingxiang Zeng	7
3	Wenlong Liu	10	8	Eli O. Meltzer	7
4	U. Wahn	8	9	Jan Sundell	6
5	Anna Maria Zicari	8	10	P. Scheinmann	6

According to the CiteSpace software, 1,618 co-cited authors have 734 cooperations in the field of “AR in Children,” as shown in Figure 3B. The most represented author is J. Bousquet (321 citations) from France, who focused on asthma and AR, followed by M.I. Asher (145 citations), E.O. Meltzer (133 citations), and J.L. Brozek (91 citations). The relationship between groups of authors was independent.

### Analysis of countries

**FIGURE 4:**
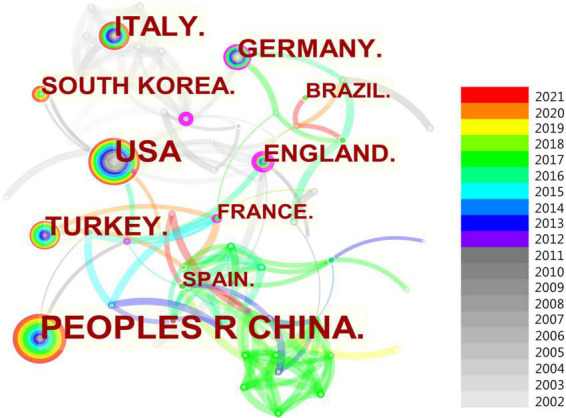
Collaborating Countries on AR research in children.

From 2002 to 2021, the 649 published papers related to AR research in children were from 100 countries (Figure 4). Of these countries, more than half have published less than 5 papers, and 470 studies were from the top 10 countries, accounting for 72.4% of all publications. The top 10 countries in terms of publication numbers are shown in Table 2. The country with the most publications was China (*n* = 139), followed by the USA (*n* = 98), Italy (*n* = 63), and Turkey (*n* = 59). The country with the highest centrality was the USA (0.38), followed by the UK (0.33), which indicates that the USA and the UK played important roles in cooperation between countries. In Figure 4, each node represents one country, and each link denotes one cooperation. There were 278 cooperation links among the countries.

**TABLE 2:** Top 10 countries in terms of the number of studies published in 2002–2021.

No.	Country	Counts	Centrality	No.	Country	Counts	Centrality
1	China	139	0	6	South Korea	38	0
2	The USA	98	0.38	7	The UK*	36	0.33
3	Italy	63	0	8	Spain	22	0.08
4	Turkey	59	0	9	Brazil	20	0
5	Germany	44	0.13	10	France	20	0.11

*The UK including England, Scotland, Wales, and Northern Ireland.

### Analysis of institutions

**FIGURE 5:**
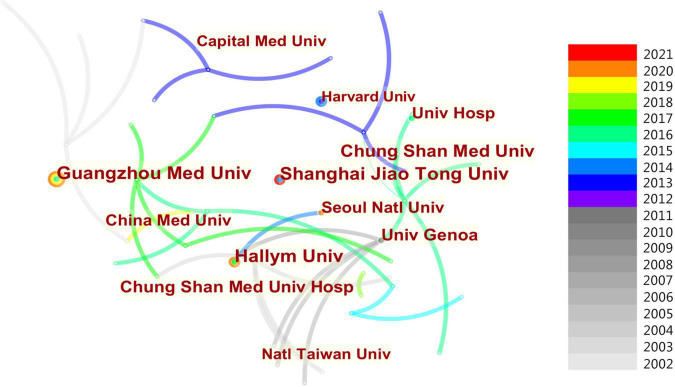
Institutions of AR research in children.

The CiteSpace software was used to analyze the 1,232 institutions that made contributions to AR research in children as shown in Figure 5. Of these, only 3.6% published more than 5 papers. Table 3 shows the top 10 institutions in terms of the number of studies published from 2002 to 2021. It can be seen that the top 10 institutions have published 124 studies, accounting for 19.1% of all publications. According to the results, Guangzhou Medical University in China and Hallym University in South Korea were the institutions with the most articles, with 17 published papers each, followed by Shanghai Jiao Tong University (16 papers), Chung Shan Medical University (16 papers) from China, and University of Genoa (13 papers) from Italy.

**TABLE 3:** Top 10 institutions in terms of the number of studies published in 2002–2021.

No.	Counts	Institution	Country/Region	Centrality
1	17	Guangzhou Medical University	China	0.01
2	17	Hallym University	Korea	0.02
3	16	Shanghai Jiao Tong University	China	0
4	16	Chung Shan Medical University	China	0.11
5	13	Genoa University	Italy	0.03
6	12	China Medical University	China	0.13
7	12	Seoul National University	Korea	0.08
8	11	National Taiwan University	China	0.02
9	10	Capital Medical University	China	0.09
10	8	Harvard University	The USA	0.09

### Analysis of journals

A total of 649 articles were published in 236 different journals. Table 4 shows the top 10 journals in the last 20 years for publishing research studies on AR in children. Among these, the most articles were published in *Pediatric Allergy and Immunology* (*n* = 65), followed by the *International Journal of Pediatric Otolaryngology* (*n* = 48), and *Annals of Allergic Asthma Immunology* (*n* = 31).

**TABLE 4:** Top 10 most popular journals in 2002–2021.

No.	Counts	Journal	Impact factor
1	65	*Pediatric Allergy and Immunology*	6.377
2	48	*International Journal of Pediatric Otorhinolaryngology*	1.675
3	31	*Annals of Allergy, Asthma, and Immunology*	6.347
4	23	*Journal of Allergy and Clinical Immunology*	10.793
5	21	*American Journal of Rhinology and Allergy*	2.467
6	21	*Allergy*	13.146
7	20	*Allergy and Asthma Proceedings*	2.587
8	19	*Allergologia ET Immunopathologia*	1.667
9	15	*Allergy Asthma and Respiratory Disease*	0.07
10	14	*International Archives of Allergy and Immunology*	2.749

**FIGURE 6:**
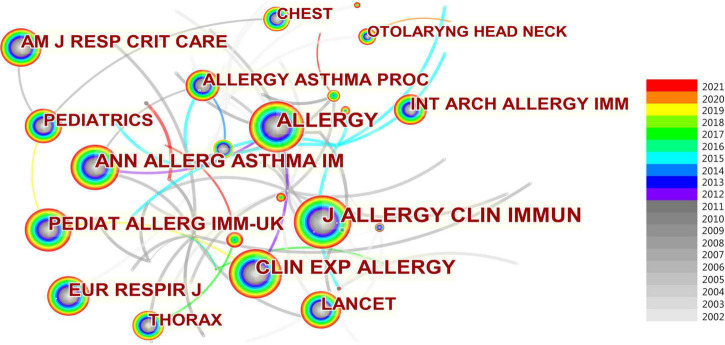
Co-cited Journals of AR research in children.

Regarding the top 10 co-cited journals in Figure 6, *The Journal of Allergy and Clinical Immunology* has the largest number of citations (580 citations) for AR research in children, followed by *Allergy* (532 citations), *Clinical and Experimental Allergy* (429 citations), *Annals of Allergy Asthma and Immunology* (343 citations), and *Pediatric Allergy and Immunology* (336 citations). And *Lancet* had the highest impact factor (79.323) in 2020 which has 234 citations. The most important journals in this field are the *Journal of Allergy and Clinical Immunology*; *Allergy*; *Allergy and Asthma Proceedings*; and *Annals of Allergy, Asthma and Immunology*, due to their forefront both in terms of publication and co-citation.

### Analysis of co-cited literatures

Co-citation in the literature can to some extent reflect the crucial literature in the field (10). Among the high-frequency co-cited documents, the top 10 articles are the core documents in this field (Table 5) and are worthy of our study. Among these articles, Erkkola et al. (16); Kuehr et al. (17), and Chervinsky et al. (18) gave examples of treatments such as maternal vitamin D intake, combination treatment with anti-IgE plus specific immunotherapy, and Omalizumab. Weidinger et al. (19), Weiland et al. (20), Braun-Fahrlander et al. (21), and Burgess et al. (22)described the epidemiology. However, most of the literature is more than 10 years old, and there is no study within the last 2–3 years.

**TABLE 5:** The top 10 references with the most co-citation counts.

No.	Counts	References	Article
1	300	Erkkola et al. (16)	Maternal vitamin D intake during pregnancy is inversely associated with asthma and allergic rhinitis in 5-year-old children
2	280	Weidinger et al. (19)	Filaggrin mutations, atopic eczema, hay fever, and asthma in children
3	250	Kuehr et al. (17)	Efficacy of combination treatment with anti-IgE plus specific immunotherapy in polysensitized children and adolescents with seasonal allergic rhinitis
4	213	Weiland et al. (20)	Climate and the prevalence of symptoms of asthma, allergic rhinitis, and atopic eczema in children
5	168	Hatzler et al. (23)	Molecular spreading and predictive value of preclinical IgE response to Phleum pratense in children with hay fever
6	155	Braun-Fahrlander et al. (21)	No further increase in asthma, hay fever, and atopic sensitization in adolescents living in Switzerland
7	152	Chervinsky et al. (18)	Omalizumab, an anti-IgE antibody, in the treatment of adults and adolescents with perennial allergic rhinitis
8	146	Burgess et al. (22)	Childhood allergic rhinitis predicts asthma incidence and persistence to middle age: A longitudinal study
9	140	Deng et al. (24)	Exposure to outdoor air pollution during trimesters of pregnancy and childhood asthma, allergic rhinitis, and eczema
10	122	De Groot et al. (25)	Allergic rhinitis is associated with poor asthma control in children with asthma

### Analysis of keywords

**FIGURE 7:**
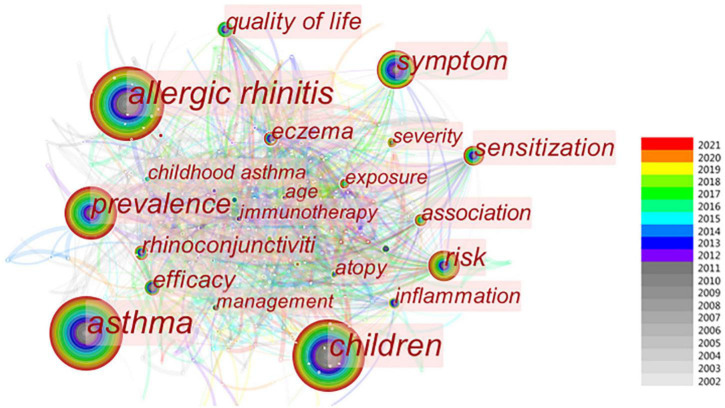
Keywords of AR research in children.

**FIGURE 8:**
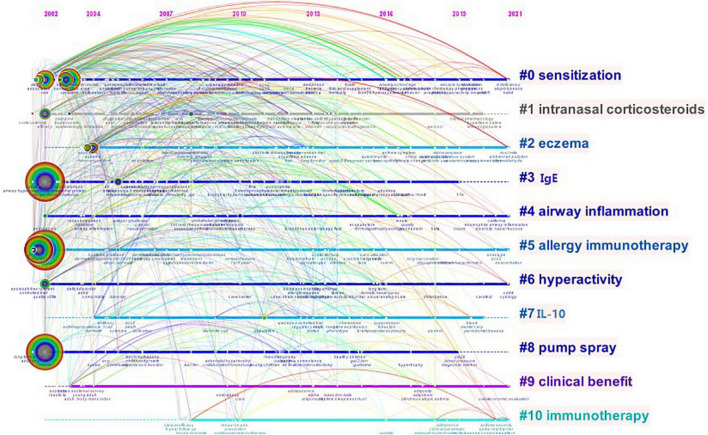
Time-line map of keyword clusters.

The keywords in the included publications were analyzed by CiteSpace (Figure 7). The co-occurrence frequency shown in Table 6 demonstrates that the keyword with the highest frequency was “AR” at 380, followed by “asthma” (338), “children” (315), “prevalence” (196), and “symptom” (121). All the keywords in this study were classified into 11 clusters (Figure 8): #0 “sensitization,” #1 “intranasal corticosteroids,” #2 “eczema,” #3 “IgE,” #4 “airway inflammation,” #5 “allergy immunotherapy,” #6 “hyperactivity,” #7 “IL-10,” #8 “pump spray,” #9 “clinical benefit,” and #10 “immunotherapy.” According to Figure 8, keyword clusters such as “sensitization,” “airway inflammation,” “allergy immunotherapy,” and “hyperactivity” appeared in 2002, and persisted for 20 years. In the years that followed, some keywords like “eczema,” “clinical benefit,” and “immunotherapy” appeared, and persist until today.

**TABLE 6:** Top 20 keywords in 2002–2021.

No.	Keyword	Counts	No.	Keyword	Counts
1	Allergic rhinitis	380	11	Eczema	46
2	Asthma	338	12	Association	44
3	Children	315	13	Inflammation	34
4	Prevalence	196	14	Atopy	29
5	Symptom	121	15	Severity	27
6	Risk	86	16	Childhood asthma	27
7	Sensitization	67	17	Exposure	27
8	Quality of life	52	18	Age	23
9	Efficacy	51	19	Management	22
10	Rhinoconjunctivitis	50	20	Immunotherapy	22

**FIGURE 9:**
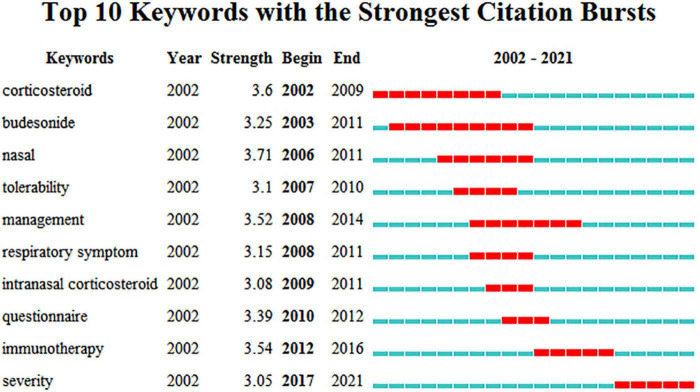
Top 10 burst keywords of AR research in Children. The blue part represents the base timeline, and the red part indicates the burst duration of the keyword. The strength represents the intensity of hotspots in clinical research of the burst keyword. A greater strength indicates a higher frequency of occurrence.

Burst keywords were detected using the CiteSpace software, which can be used as indicators of development and emerging trends. The top 10 keywords with the strongest citation bursts in the field are shown in Figure 9, and the burst strength and timespan varied. The burst keyword with the highest strength was “nasal” (strength = 3.71), which lasted for five consecutive years from 2006 to 2011, followed by “corticosteroid” (strength = 3.6, 2002–2009), and “immunotherapy” (strength = 3.54, 2012–2016). The burst keyword “severity” (strength = 3.05) has received increased attention in recent years.

## Discussion

In recent years, many scholars have been committed to studying AR in children, and considerable research achievements have been made in this field so far. However, AR in children is still a thorny global problem. The number of publications has continued to increase steadily and rapidly over the last 20 years. Many meta-analyses and systematic reviews were conducted on AR in children. And, it demonstrated that we have a lot of experience in treating AR in children (26, 27).

Since there are few articles in the previous period, only literature in the past 20 years was selected for statistical analysis. In total, we selected 649 relevant publications from the WOS Collection. All the studies were published by 3,299 authors from 100 different countries. Giorgio Ciprandi from Italy is the most influential author in this field with the most literature. China has the highest number of publications (*n* = 139) and the most connections with other countries. The countries with the highest centrality were the USA and the UK. Guangzhou Medical University in China and Hallym University in Korea were the most productive institution. Through the visualization analysis, we can conclude that China, the USA, and France contributed the most to AR research. Unfortunately, there is not enough cooperation among them at present. Most of these papers were published in journals in pediatrics, otolaryngology, or immunology, such as *Pediatric Allergy and Immunology* and the *International Journal of Pediatric Otolaryngology. Journal of Allergy and Clinical Immunology* has the largest number of citations.

Keywords summarize the article and can embody the important content of the article. Keyword clusters can classify the degree of similarity between indicators. Therefore, keywords and clusters can serve as important indexes to reflect the research hotspots and international focus of the literature in a certain field. The keyword with the highest frequency was “AR,” followed by “asthma,” and “children.” The burst keywords with the highest strength were “nasal,” “corticosteroid,” and “immunotherapy.” The keyword “severity” is prevalent from 2017 to today. Based on the cluster analysis of keywords, the research pattern of AR in the past 20 years and the research trend in the future are further explored. According to these results, we comprehensively analyzed the clusters and identified the following top hotspots in research on AR in children: pathogenesis (atopy, air pollution, eosinophils, sensitization, tolerability, pollen, and hyperactivity), comorbidity (asthma, eczema, rhinoconjunctivitis, non-AR, and airway inflammation), epidemiology (prevalence and questionnaire), symptom (nasal, symptom score, respiratory symptom, and severity), and therapy (budesonide, cetirizine, loratadine, intranasal corticosteroid, nasal spray, and sublingual immunotherapy). At present, a very important component of the clinical treatment of AR is to avoid exposure to allergens and various irritants, but this is often difficult to achieve in real life. Therefore, drug therapy, such as budesonide, cetirizine, loratadine, and intranasal corticosteroid, remains the primary means, based on visual analysis, of improving the quality of life of patients. However, the bioavailability of nasal glucocorticoids is low, and the effect on the growth and development of children is not clear. Some children fail to adhere to continuous medication treatment because their parents worry about the adverse reactions of drugs, such as nasopharyngeal discomfort.

In 2008, Wallace et al. (28) summarize the diagnosis of AR, including typical examination findings such as allergic shiners, Dennie-Morgan lines, allergic salute, allergic facies, nasal mucosa, and cobblestoning. The study suggests that determining specific IgE positivity along with the clinical symptoms and physical examination can confirm the diagnosis. Bousquet et al. (29) state that AR in children can be classified according to whether it is intermittent or persistent as well as based on severity. With the development of AR research, therapy was becoming more standardized. Schuler and Montejo (30) conclude that the therapy for AR in children can be conceptualized as a 3-pronged approach including avoidance, medications (including nasal corticosteroid), and immunotherapy (including sublingual immunotherapy).

Immunotherapy was defined as “therapeutic vaccination” (31) by WHO in 1998. The effectiveness of immunotherapy in adults and children who were allergic to house dust mites was demonstrated by Purello-D’Ambrosio et al. (32) in 2001. Therefore, immunotherapy has not only been shown to improve AR by reducing the severity of the disease but also may prevent further allergic sensitizations and asthma development. The most widely used are subcutaneous injections and sublingual immunotherapy at this time. In 2000, Ross et al. found that 3–5 years of subcutaneous injections are the most effective (33). However, it is difficult for children to continue this treatment. Sublingual immunotherapy is easier to accept for children. But, because of the lack of available sublingual immunotherapy products for children, it is less relevant for pediatric patients. In the future, appropriate sublingual immunotherapy products may be invented for children.

Recently, the severity of AR in children has been taken seriously and is part of mechanism studies. A study by Di Cara et al. (34) indicates that the severity of AR may be an important factor in increasing the risk of asthma development in children. Some indices were found to relate to the severity of AR. In 2008, Rolinck-Werninghaus et al. (35) discovered that the baseline specific IgE is associated with symptom severity during the pollen season in children with SAR. In 2013, Ozkaya et al. (36) proposed that plasma paraoxonase activity and oxidative stress levels may serve as predictors of disease severity in children with AR. A 5-year study found that the presence of adenoid hypertrophy increases the severity of the disease (37). Researchers discovered that nasal septum deformity, imbalance of effector T cells and regulatory cells, and the prolongation of mucociliary clearance (38–40) increase AR severity.

A few limitations of this visualization analysis must be considered. First, our findings in this study may not be comprehensive because of limited literature. All of our publications came from the WOS Core Collection database, and articles published in other ways were excluded. Second, the CiteSpace software does not clearly distinguish the first author from the corresponding author. Third, the top 50 authors, countries, institutions, and keywords were selected as the selection strategy, which may have some bias. However, we argue that this study can be used to describe the hotspot and emerging trends in this field.

## Conclusion

According to the results, we conclude that the hotspots are therapy and mechanism research of AR in children. The frontiers of AR research in children mainly include immunotherapy and severity analysis. China is the leading country in AR research. However, the USA and France also play a role in promoting the development of this research field. Further clinical trials and more rigorous research related to AR in children are needed in the future which contribute to the possible prevention and even curing of AR and benefit patients in the future.

## Author contributions

YL conceived and designed the study. NC, RW, and LZ analyzed the data. NC and FL wrote the manuscript. All authors read and approved the final manuscript.
